# Congruency Sequence Effects without Feature Integration or Contingency Learning Confounds

**DOI:** 10.1371/journal.pone.0102337

**Published:** 2014-07-14

**Authors:** James R. Schmidt, Daniel H. Weissman

**Affiliations:** 1 Department of Experimental Clinical and Health Psychology, Ghent University, Ghent, Belgium; 2 Department of Psychology, University of Michigan, Ann Arbor, Michigan, United States of America; University of California, Merced, United States of America

## Abstract

The congruency effect in distracter interference (e.g., Stroop) tasks is often reduced after incongruent trials, relative to congruent trials. It has been proposed that this congruency sequence effect (CSE) results from trial-by-trial adjustments of attention, which are triggered by changes in response conflict, expectancy, or negative affect. Hence, a large literature has developed to investigate the source(s) of attention adaptation in distracter interference tasks. Recent work, however, suggests that CSEs may stem from feature integration and/or contingency learning processes that are confounded with congruency sequence in the vast majority of distracter interference tasks. By combining an established method for measuring CSEs in the absence of these learning and memory confounds with a prime-probe task, we observed robust CSEs in two experiments. These findings provide strong evidence of CSEs independent of learning and memory confounds, which might be explainable by trial-by-trial adjustments of attention. They also reveal a highly effective approach for observing CSEs independent of the typical confounds, which will facilitate future studies of how people adapt to distraction.

## Introduction

Distracter interference tasks are widely employed to investigate selective attention. In such tasks, participants are instructed to identify a relevant item in the presence of one or more distracters that engender either the same response as the relevant item (congruent trials) or a different response (incongruent trials). The most common distracter interference tasks are the Stroop, flanker, and Simon tasks. In the Stroop task [1], participants are asked to identify the ink color in which a word is presented (e.g., red) independent of the word's identity (e.g., RED in congruent trials, BLUE in incongruent trials). In the flanker task [2], participants are asked to identify the central letter of a three-letter array (e.g., HHH or SHS) independent of two flanking letters (e.g., two Hs in congruent trials, two Ss in incongruent trials). In the Simon task [3], participants are asked to identify a relevant item's color by making a lateralized response (e.g., left) independent of the item's spatial location (e.g., left in congruent trials, right in incongruent trials). A ubiquitous finding in such tasks is that performance is worse in incongruent than in congruent trials. This “congruency,” or “interference,” effect indicates that selective attention often fails to suppress irrelevant stimuli.

Nonetheless, the congruency effect varies considerably with task context. For example, the congruency effect is smaller on the current trial when the previous trial was incongruent, relative to congruent, as indexed by an interaction between previous- and current-trial congruency [4]–[5]. This *congruency sequence effect (CSE)*, also known as *conflict adaptation* or the *Gratton effect*, has come to occupy a central position in studies investigating the influence of task context on behavioral performance in distracter interference tasks. However, the exact process or processes that give rise to CSEs remain highly controversial.

### Competing accounts of CSEs

One class of explanation posits that CSEs index trial-by-trial adjustments of attention. Within this class, various “attention adaptation” accounts propose that stimulus congruency in the current trial influences control processes that determine the distribution of attention and/or effort to targets and distracters in the next trial. The conflict monitoring account posits that heightened levels of response conflict in an incongruent trial trigger control processes to increase attention to task-relevant stimuli, and/or suppress attention to task-irrelevant stimuli, in the next trial [6]. The expectation account posits a mechanism that deploys more or less attention to targets and/or distractors in the next trial as a function of whether current-trial distracter processing impairs or aids performance [5]. In particular, based on an expectation that distracter processing will influence performance similarly in the next trial, more attention to the target and/or less attention to the distracter is deployed after an incongruent trial while the opposite occurs after a congruent trial. The negative affect account posits that an incongruent trial primes negative affect, which triggers control processes to increase attention and/or effort in the next trial [7]–[8]. All of these accounts posit a relative shift of attention toward the target and away from the distracter after an incongruent trial (relative to a congruent trial) that reduces the size of the congruency effect. Not surprisingly, a great deal of effort has been expended to distinguish among these and other “attention adaptation” accounts of CSEs.

Another class of explanation – the “learning and memory” account – does not rely on the idea of an attention adaptation mechanism, but instead posits that CSEs index learning and memory processes, which are confounded with congruency sequence in the vast majority of distracter interference tasks. The feature integration account posits that CSEs stem from unequal repetitions of stimulus and/or response features across different congruency sequences [9]–[10]. For example, in this view, the congruency effect is greater after congruent than after incongruent trials (at least in part) because exact stimulus repetitions that speed responses occur only in (a) congruent trials preceded by congruent trials (which increases the congruency effect after congruent trials) and (b) incongruent trials preceded by incongruent trials (which reduces the congruency effect after incongruent trials).

Another instance of a learning and memory account – the contingency learning account – posits that CSEs stem from associating a distracter with the congruent response more often than with any particular incongruent response [11], a typical consequence of equating the number of congruent and incongruent trials in tasks involving more than two possible stimuli and responses [12]–[13]. Strengthening the association between a distracter and the congruent response speeds responses in congruent trials, and this phenomenon is called the *contingency* effect. Moreover, the contingency effect is larger following a *high-contingency* trial in which the distracter is presented with the most frequent target than after a *low-contingency* trial in which the distracter is presented with a less frequent target [14]. That is, the difference in mean response time between (high contingency) congruent and (low contingency) incongruent trials is increased following a (high contingency) congruent trial relative to a (low contingency) incongruent trial due to the previous by current *contingency* interaction that confounds the previous by current *congruency* interaction. While the precise mechanism underlying this *sequential contingency* effect remains unknown (although temporal learning provides one potential explanation [15]), it is clear that when congruency is confounded with contingency CSEs may reflect sequential contingency effects, rather than some form of attention adaptation. Lending credence to the learning and memory class of explanation, some findings indicate that CSEs in the Stroop, flanker, and Simon tasks are eliminated after controlling for feature integration and contingency learning confounds [11], [16], suggesting that CSEs do not necessarily stem from trial-by-trial adjustments of attention in these tasks.

### CSEs without learning and memory confounds?

There is, however, some evidence to suggest that trial-by-trial adjustments of attention might contribute to CSEs in the prime-probe arrow task. Here, one or more distracter arrows pointing left, right, up, or down are presented just prior to a target arrow which also points left, right, up, or down. The task is to indicate the direction in which the target arrow points. Notably, even after removing trials with feature repetitions, CSEs were observed in versions of this task that contained no contingency learning confounds [17]. However, since leftward, rightward, upward, and downward pointing arrows are all mental rotations of the same stimulus, stimulus repetitions coupled with a mental rotation strategy could account for CSEs in this task. For example, Kunde and Wuhr [17] suggested that subjects might mentally rotate the current-trial target to match the orientation of the target from the previous trial.

To test this hypothesis, Kunde and Wuhr [17] assessed performance as a function of the orientation disparity (90°, 180°, 270°) between the current-trial target and the previous-trial target. While they found no evidence to support a mental rotation strategy specific to the target stimuli, they did not conduct additional analyses to test whether (a) the current-trial target was mentally rotated to match the orientation of the previous-trial distracter, (b) the current-trial distracter was rotated to match the orientation of either the previous-trial target or the previous-trial distracter, or (c) both the current-trial target and the current-trial distracter were rotated to match the previous-trial target and previous-trial distracter, either individually or as part of a single integrated percept. Thus, while it is unclear whether adopting such complex strategies would have aided performance, the possibility that CSEs in the prime-probe arrow task were mediated by stimulus repetitions coupled with a mental rotation strategy was not completely ruled out. Experiment 1 of the present article controlled for the first two mental rotation strategies above by ensuring that the target and distracter in each trial were always ±90° rotations of the target and distracter in the previous trial. Experiment 2 controlled for all three mental rotation strategies above by employing a paradigm in which such strategies were not possible.

Like many other investigations of CSEs that have employed four-alternative-forced choice (4-AFC) tasks, Kunde and Wuhr's [17] experimental design had two additional limitations. First, pairing each of the four possible distracters with each of the four possible targets equally often to avoid contingency biases [11], [16] resulted in a 4-AFC task with only 25% congruent trials. It has been suggested that using a low proportion of congruent trials might encourage subjects to focus attention on all trials [18], which might reduce the probability of observing CSEs. However, we are not aware of convincing evidence to support this notion, and some findings even suggest against it by showing that effects due to the proportion of congruent trials and CSEs are dissociable (each can be observed in the absence of the other) and generally uncorrelated [19]–[21]. Second, because all possible distracters were paired equally often with all possible targets, a large number of trials with feature repetitions needed to be discarded prior to the analyses of CSEs [16]. This procedure may have reduced the statistical power of the study. Although Kunde and Wuhr observed CSEs despite these limitations, other researchers may have been unsuccessful because of them.

### The present approach to investigating CSEs

In the present study, we used an established method for overcoming both of these limitations [10], [22]–[24]. First, the method employs a 50–50 mix of congruent and incongruent trials in 4-AFC distracter interference tasks without introducing contingency learning confounds. This can be accomplished in a prime-probe arrow task by pairing each distracter arrow (e.g., “<”) with one congruent target arrow (e.g., “<”) and one incongruent target arrow (e.g., “>”) from the same orientation category (i.e., horizontal or vertical), rather than with all possible target arrows (i.e., “<”, “>”, “⁁”, and “∨”). The result is four congruent target-distracter pairs (< <, > >, ⁁ ⁁, ∨ ∨) and four incongruent target-distracter pairs (< >, > <, ⁁ ∨, ∨ ⁁), each of which contains arrows from only the horizontal or only the vertical orientation category. This procedure may result in a certain amount of contingency learning, because each distracter is linked to only two of the four possible targets. However, any such learning should be equivalent for congruent and incongruent trials, because each distracter is linked to one congruent target and one incongruent target (i.e., each distracter arrow is equally predictive of one congruent response and one incongruent response). Thus, congruency is not confounded with contingency, unlike in the vast majority of 4-AFC distracter interference tasks [11], [16]. Second, the method avoids feature repetitions without discarding trials. This can be accomplished in a prime-probe arrow task by presenting target-distracter pairs made of leftward and rightward pointing arrows in odd-numbered trials and target-distracter pairs made of upward and downward pointing arrows in even-numbered trials. This alternation, which is independent of both previous-trial and current-trial congruency (and, hence, CSEs), ensures the complete absence of first-order feature repetitions in the trial sequence.

As mentioned earlier, to our knowledge only a few prior studies have employed the method above for investigating CSEs without feature integration and contingency learning confounds, albeit none with the prime-probe task [10], [22]–[24]. Of importance, many of these studies failed to observe CSEs. First, Mayr and colleagues [10] did not observe a CSE in an arrow flanker task. Second, Jiménez and Mendez [22] did not observe a first-order CSE in a Stroop task (they did, however, observe higher-order CSEs from trials *n* – 2 and *n* – 3). Third, Lee and Cho [24] did not observe a CSE when participants alternated between vertical and horizontal Simon tasks (Experiment 1a) or spatial Stroop tasks (Experiment 1b). These findings are consistent with Schmidt and De Houwer's [11] argument that the CSE is due mainly to feature repetition and contingency learning biases.

Lee and Cho [24], however, suggested the CSE might be observed when both the task-irrelevant dimension and the “response mode” repeat on consecutive trials. While the experiments they conducted to test this hypothesis did not completely control for feature repetition and contingency learning confounds, a follow-up study by Kim and Cho [23] observed support for this hypothesis in an experiment that *did* control for these confounds. In this study, the researchers divided a 4-AFC color flanker task into a pair of 2-AFC S-R mappings that involved two distinct colors and two distinct responses. They then alternated between trials involving these two S-R mappings. As hypothesized, the researchers observed a CSE when each of the four possible responses was made with the right hand but not when two responses were made with the right hand and two responses were made with the left hand. They therefore concluded that observing a CSE depends upon participants perceiving two tasks as involving the same “response mode” (i.e., hand). They further concluded that a previous failure to observe a CSE when participants alternated between horizontal and vertical flanker tasks involving the same response mode [10] occurred because horizontal and vertical stimuli are processed by different brain mechanisms and thus involve distinct conflict detection and control systems.

### Goals of the present study

In sum, while the vast majority of findings to date suggest the CSE is simply an artifact of feature repetition and contingency learning confounds, some data suggest the CSE might exist independent of these confounds in the prime-probe arrow and color flanker tasks. However, prior findings from the prime-probe task are inconclusive due to mental rotation confounds, and prior data from the color flanker task suggest that two tasks must share common brain mechanisms and response modes for a CSE to emerge. Since it is less certain whether a CSE can be observed independent of the typical learning and memory confounds in the prime-probe task than in the color flanker task, we focused on the prime-probe task in the present study. In light of Kim and Cho's [23] findings, however, we investigated whether a CSE can be observed without the typical confounds in the prime-probe task even when participants alternate between horizontal and vertical prime-probe tasks that involve different “response modes” (i.e., hands), as was the case in Kunde and Wuhr's [17] study. Thus, we investigated not only whether it is possible to observe a CSE independent of the typical confounds in the prime-probe task, but also whether the CSE can be observed under a considerably less restrictive set of task conditions in the prime-probe task than in the color flanker task.

## Experiment 1

The goal of Experiment 1 was to conceptually replicate Kunde and Wuhr's [17] finding of CSEs in the prime-probe arrow task using the method above for avoiding feature integration and contingency learning confounds. In each trial of Kunde and Wuhr's task, a relatively small distracter arrow preceded a relatively large target arrow. Here, we presented five distracter arrows rather than just one to increase overall levels of interference [25]. Beyond this modification, however, the two experiments were highly similar. We therefore reasoned that conceptually replicating Kunde and Wuhr's finding of CSEs in the prime-probe arrow task would serve to validate the method above for investigating CSEs.

### Methods

#### Ethical Statement

This study was approved by the University of Michigan Behavioral Sciences Institutional Review Board. Each participant gave informed written consent before starting the experiment.

#### Participants

Sixteen young adults (mean age: 21.2 years; age range: 18–27 years; 12 male) from the Ann Arbor community were recruited via flyers posted on the University of Michigan campus. The flyers stated that participants should be 18–30 years of age and have normal or corrected-to-normal vision. They also stated that individuals could not participate if they had ever suffered a serious head injury, had a history of seizures, or if they were taking prescription or recreational psychoactive drugs. When each person arrived at the laboratory, he or she first provided informed written consent to participate in the study as required by the University of Michigan's Institutional Review Board. Individuals were then screened to ensure that they met the inclusion criteria above. Each person was paid $10 per hour for participating in the study, which took about an hour.

#### Stimuli

A fixation cross (0.8°×0.8°) appeared at the center of the screen for two seconds at the beginning of each block. Each trial contained three sequential events presented at the center of the screen: a distracter array (133 ms), a blank screen (33 ms), and a target (133 ms). The distracter array in the current trial always appeared 2,000 ms after the onset of the distracter array in the previous trial. The distracter array contained five arrows (each 1.04°×1.04°) pointing in the same direction (left, right, up, or down). Distracter arrays made of leftward (“<”) or rightward (“>”) pointing arrows were oriented horizontally (6.22°×1.04°) while those made of upward (“⁁”) or downward (“∨”) pointing arrows were oriented vertically (1.04°×6.22°). The target (1.56°×1.56°) was a leftward, rightward, upward, or downward pointing arrow. By combining each of the four distracter arrays with each of two targets from the same horizontal or vertical array (e.g., “>>>>>” was only presented with either “>” or “<”), we created eight unique distracter-target pairings. Four pairings were congruent (left-left, right-right, up-up, down-down) and four were incongruent (left-right, right-left, up-down, down-up).

#### Design

Each participant completed a single practice block containing 24 trials and eight test blocks containing 96 trials each. The trials in each test block were presented in a pseudo-randomized order. This order equated the number of trials in each congruency sequence. In particular, there were 24 congruent trials preceded by congruent trials (cC trials), 24 incongruent trials preceded by congruent trials (cI trials), 24 congruent trials preceded by incongruent trials (iC trials), and 24 incongruent trials preceded by incongruent trials (iI trials). To avoid feature integration confounds, target-distracter pairs made of leftward and rightward pointing arrows were presented in odd-numbered trials while target-distracter pairs made of upward and downward pointing arrows were presented in even-numbered trials. To avoid contingency learning confounds, each target-distracter pair occurred exactly twelve times in every test block.

#### Procedure

Participants were told to indicate whether the target arrow in each trial pointed left, right, up, or down as quickly as possible without making mistakes. To do so, they used their left middle finger, left index finger, right middle finger, or right index finger, respectively, to press the F (left), G (right), J (up), or N (down) key. The word “Error” was presented at the center of the screen for 200 ms after each incorrect response and after each response omission (i.e., trials in which no response was made within 1,500 ms of target onset). We employed Psychtoolbox [26] on a Windows PC to present the stimuli and to record participants' responses.

#### Data analysis

Practice trials were not analysed. Prior to analyzing each participant's response time (RT) data, errors (4.0% of the data), trials immediately following errors (3.5% of the data), response omissions (0.38% of the data), trials immediately following response omissions (0.36% of the data), and RT outliers among the remaining trials (i.e., trials with RTs greater than three standard deviations from the conditional mean; 1.5% of the data) were discarded. The same trials were discarded prior to analyzing each participant's error rate data, with the exception of errors (since errors were the dependent measure in this analysis). Subsequently, mean correct RT and mean percentage error rate were calculated in each participant for the four congruency trial sequences in the experiment: cC, cI, iC, and iI. Repeated-measures analyses of variance (ANOVAs) were used to evaluate the significance of both overall congruency effects and CSEs.

### Results

#### Response times

**Figure 1:**
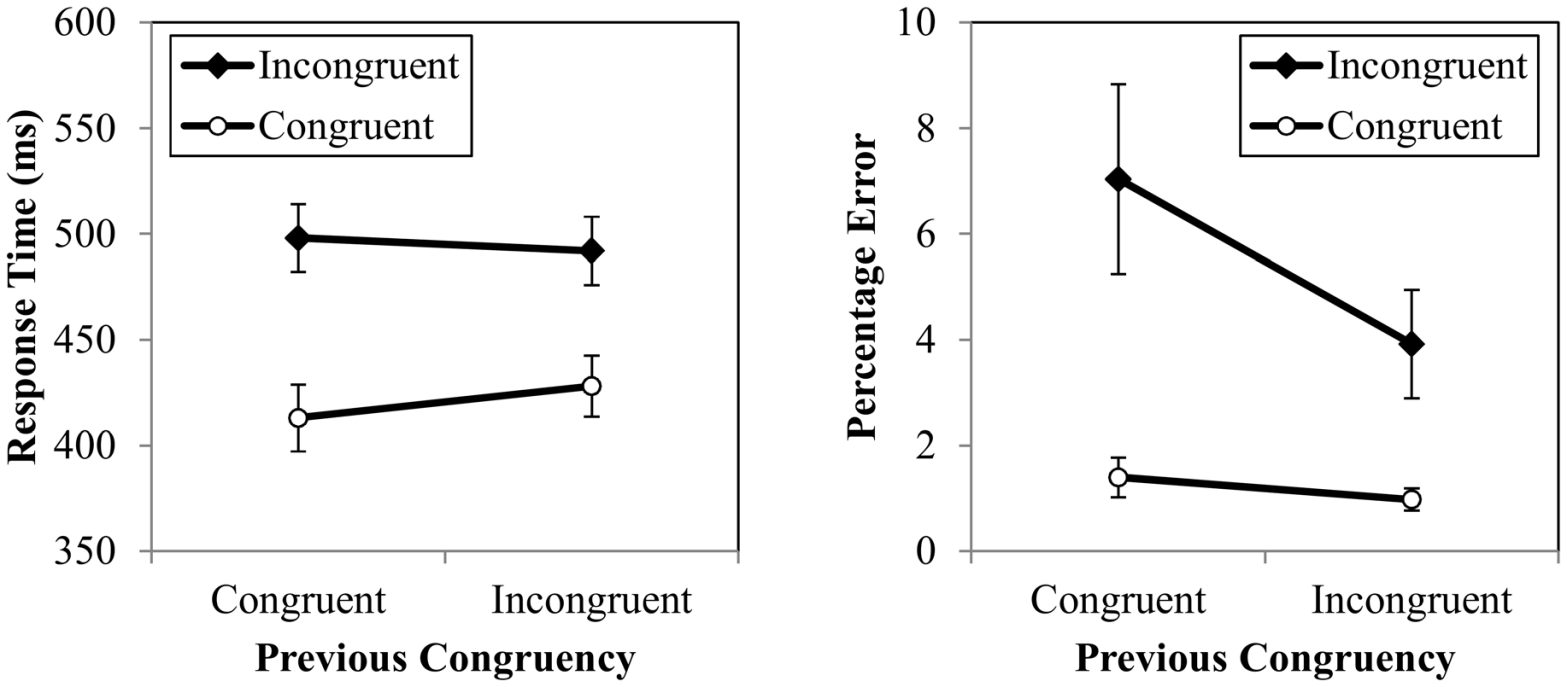
Experiment 1 response times (left) and percentage errors (right) for previous and current congruency. Error bars represent one standard error from the cell mean.

The response time results for Experiment 1 are presented in Figure 1. Mean RT was analyzed using a repeated measures analysis of variance (ANOVA) with two factors: previous congruency (congruent vs. incongruent) and current congruency (congruent vs. incongruent). As expected, there was a main effect of current congruency, *F*(1,15) = 144.363, *p*<.001, 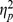 = .91, indicating slower RTs in incongruent trials (495 ms) than in congruent trials (421 ms). There was also a main effect of previous congruency, *F*(1,15) = 13.124, *p* = .003, 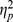 = .47, resulting from faster RTs when the previous trial was congruent (456 ms) as compared to incongruent (460 ms). Finally, as expected, there was an interaction between previous congruency and current congruency, *F*(1,15) = 25.967, *p*<.001, 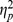 = .63, indicating less interference after incongruent trials (64 ms) than after congruent trials (84 ms). As expected, tests of simple effects revealed that mean RT was longer in cI trials (498 ms) than in iI trials (492 ms), *t*(15) = 2.234, *p* = .041, *η* ^2^ = .54, and longer in iC trials (428 ms) than in cC (413 ms) trials, *t*(15) = 6.436, *p*<.001, *η* ^2^ = .73. No other effects were significant.

We also investigated whether CSEs were influenced by trial *n* – 2 feature repetitions. To do so, we conducted a repeated-measures ANOVA on mean RT using three within-participants factors: previous congruency (congruent, incongruent), current congruency (congruent, incongruent), and trial *n* – 2 feature repetitions (complete repetitions, complete alternations, target repetitions, distracter repetitions). Replicating the overall analysis, there was a significant main effect of current congruency, *F*(1,15) = 120.465, *p*<.001, 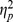 = .89, no significant main effect of previous congruency, *F*(1,15) = .001, *p* = .974, 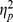
<.01, and a significant interaction between previous congruency and current congruency, *F*(1,15) = 37.466, *p*<.001, 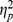 = .71. New to the present analysis, the main effect of trial *n* – 2 feature repetitions was significant, *F*(3,45) = 5.163, *p* = .004, 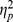 = .26, as was the interaction between trial *n* – 2 feature repetitions and current congruency, *F*(3,45) = 4.604, *p* = .007, 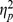 = .23. However, there was neither a two-way interaction between trial *n* – 2 feature repetitions and previous congruency, *F*(3,45) = .789, *p* = .506, 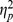 = .05, nor a three-way interaction among trial *n* – 2 feature repetitions, previous congruency, and current congruency, *F*(3,45) = 1.298, *p* = .287, 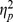 = .08. Thus, trial *n* – 2 feature repetitions did not influence CSEs.

#### Percentage errors

The percentage error rate results are also presented in Figure 1. Mean percentage error rate was analyzed using a repeated measures analysis of variance (ANOVA) with two factors: previous congruency (congruent vs. incongruent) and current congruency (congruent vs. incongruent). An expected, there was a main effect of current congruency, *F*(1,15) = 12.816, *p* = .003, 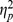 = .46, because mean percentage error rate was higher in incongruent trials (5.48%) than in congruent trials (1.19%). There was also a main effect of previous congruency, *F*(1,15) = 10.027, *p* = .006, 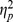 = .40, because mean percentage error rate was lower when the previous trial was incongruent (2.45%) as compared to congruent (4.22%). Finally, there was a significant interaction between previous congruency and current congruency, *F*(1,15) = 11.323, *p* = .004, 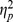 = .43, because there was less interference after incongruent trials (2.94%) than after congruent trials (5.64%). As expected, tests of simple effects revealed that mean percentage error rate was higher in cI trials (7.04%) than in iI trials (3.92%), *t*(15) = 3.407, *p* = .004, *η* ^2^ = .44. However, mean percentage error rate did not differ between iC trials (0.98%) and cC trials (1.40%), *t*(15) = 1.271, *p* = .223, *η* ^2^ = .10. No other effects were significant.

### Discussion

The results of Experiment 1 conceptually replicate Kunde and Wuhr's [17] finding that CSEs are present in the prime-probe arrow task. They also validate our methodology for investigating CSEs independent of feature repetition and contingency learning confounds. More broadly, unlike prior findings from the Stroop, flanker, and Simon tasks indicating that CSEs are eliminated after controlling for such confounds [11], [16], the present findings are encouraging for the class of accounts suggesting that some form of attention adaptation may contribute to CSEs in the prime-probe arrow task. There are still some non-attention accounts that could explain the current results (see General Discussion), but our paradigm appears to rule out simple non-attention accounts based on feature repetition and contingency learning processes. Before drawing a firm conclusion on this latter point, however, we conducted an additional experiment to rule out the possibility that a more subtle type of feature repetition confound could explain the CSEs we observed in Experiment 1.

## Experiment 2

The goal of Experiment 2 was to rule out a potential feature repetition confound in Experiment 1. As in Kunde and Wuhr's [17] study, in Experiment 1 the arrow stimuli were all rotated versions of the same stimulus. Thus, as described in the Introduction, CSEs in Experiment 1 could index feature repetitions combined with a mental rotation strategy. If the target and distracter were perceived as individual stimuli, then this strategy would be relatively easy to implement on cC and iI trials, wherein the current-trial target (e.g., “<”) and distracter (e.g., “<”) could each be rotated ±90° in the same direction to match the orientation of the previous-trial target (e.g., “⁁”) and distracter (e.g., “∨”). In contrast, this strategy would be relatively hard to implement on cI and iC trials wherein the current-trial target (e.g., “<”) and distracter (e.g., “<”) would need to be rotated ±90° in opposite directions to match the orientation of the previous-trial target (e.g., “∨”) and distracter (e.g., “⁁”). Thus, feature repetitions coupled with a mental rotation strategy could lead to worse performance on cI and iC trials, relative to cC and iI trials, mimicking the effects of attention adaptation.

On a related note, it would also be easier to implement a mental rotation strategy on cC and iI trials than on cI and iC trials if the target and distracter were perceived as an integrated stimulus that could be rotated as a unit. In this scenario, any congruent target-distracter pair (e.g., “< <”) could be rotated to match the orientation of any other congruent target-distracter pair (e.g., “⁁⁁”) but not of any incongruent target-distracter pair (e.g., “⁁ ∨”). Analogously, any incongruent target-distracter pair (e.g., “< >”) could be rotated to match the orientation of any other incongruent target-distracter pair (e.g., “⁁ ∨”) but not of any congruent target-distracter pair (e.g., “> >”). Thus, once again, feature repetitions coupled with a mental rotation strategy could lead to worse performance on cI and iC trials, relative to cC and iI trials.

While it is unclear whether adopting such complex mental rotation strategies would aid performance, the goal of Experiment 2 was to remove this potential confound. We therefore replaced the target and distractor arrows in Experiment 1 with the corresponding direction words. More concretely, we replaced “<” with “Left”, “>” with “Right”, “⁁” with “Up”, and “∨” with “Down.” It is impossible to rotate one direction word (e.g., “Left”) so that it comes to resemble another direction word (e.g., “Down”). Thus, we reasoned that observing a CSE in this prime-probe “word” flanker task would rule out the possibility that CSEs in this task are due solely to feature repetitions combined with a mental rotation strategy. Stimuli aside, Experiment 2 was an exact replication of Experiment 1.

### Methods

#### Ethical Statement

This study was approved by the University of Michigan Behavioral Sciences Institutional Review Board. Each participant gave informed written consent before starting the experiment.

#### Participants

Sixteen young adults (mean age, 21.0; age range, 18–30; 11 male) from the Ann Arbor community were recruited and paid for their participation as described in Experiment 1. None of the participants in Experiment 2 took part in Experiment 1.

#### Stimuli

In place of arrows, four direction words served as targets: “Left” (5.19°×1.25°), “Right” (6.63°×1.25°), “Up” (2.60°×1.25°), and “Down” (5.19°×1.25°). Analogously, the distracter arrow arrays from Experiment 1 were replaced with distracter word arrays, each of which contained three identical direction words stacked vertically. The height of each word in the distracter array was 0.729°. The height of the entire distracter array was 3.32°. The width of each distracter array was the width of a single word and thus varied with whether the three vertically-stacked words in the array were “Left” (3.12°), “Right” (4.16°), “Up” (1.56°), or “Down” (3.12°).

#### Design

The design was identical to that in Experiment 1.

#### Procedure

The procedure was identical to that in Experiment 1, with the exception that, in each trial, participants indicated the identity of a target word, rather than the identity of a target arrow.

#### Data analysis

The data analysis was identical to that in Experiment 1, with errors (4.17% of the data), trials following errors (3.6% of the data), response omissions (0.68% of the data), trials following response omissions (0.67% of the data), and RT outliers among the remaining trials (1.5% of the data) removed prior to analyzing each participant's RT data. Also as in Experiment 1, the same trials were removed prior to analyzing each participant's percentage error rate data, with the exception of errors (since errors were the dependent measure in this analysis).

### Results

#### Response times

**Figure 2:**
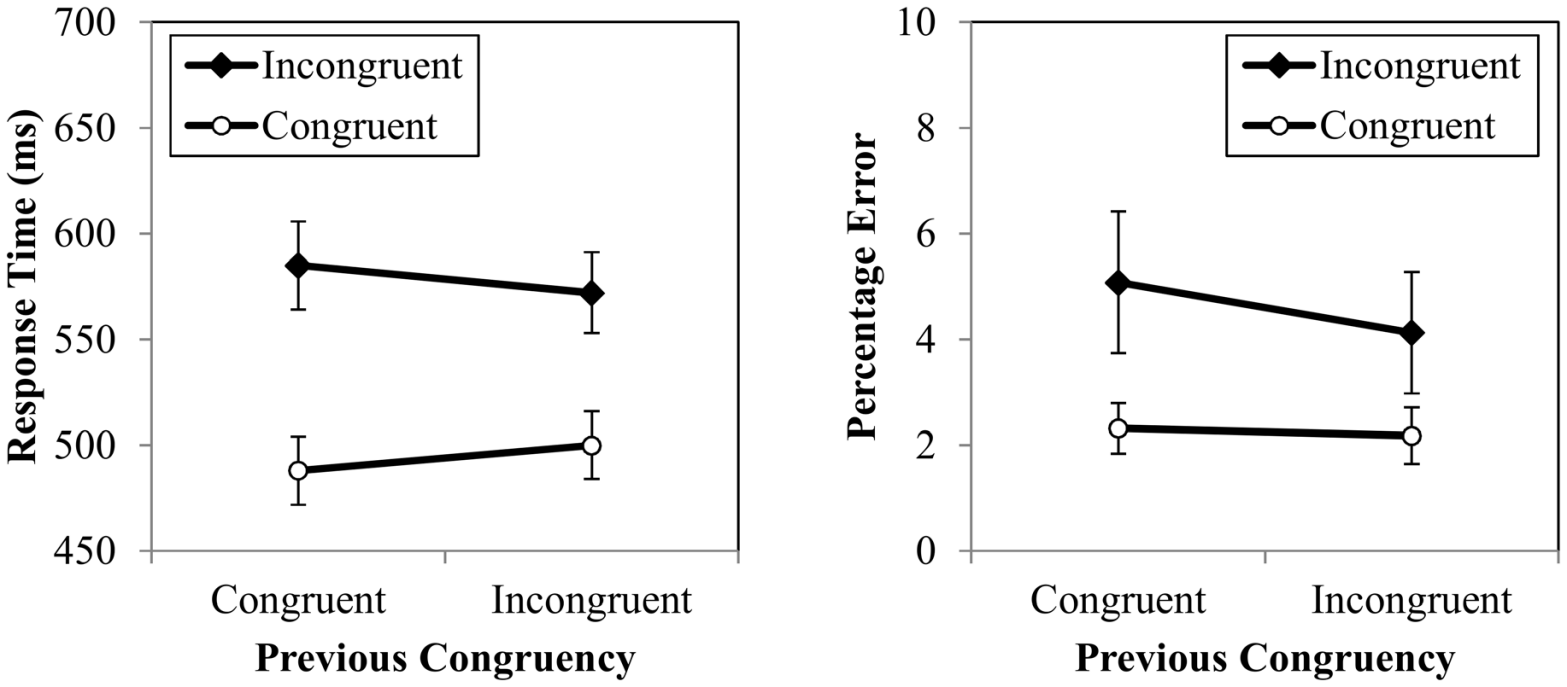
Experiment 2 response times (left) and percentage errors (right) for previous and current congruency. Error bars represent one standard error from the cell mean.

The response times for Experiment 2 are presented in Figure 2. Mean RT was analyzed using a repeated measures analysis of variance (ANOVA) with two factors: previous congruency (congruent, incongruent) and current congruency (congruent, incongruent). As expected, there was a main effect of current congruency, *F*(1,15) = 120.432, *p*<.001, 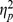 = .89, indicating that mean RT was slower in incongruent trials (578 ms) than in congruent trials (494 ms). Unlike in Experiment 1, however, the main effect of previous congruency was not significant, *F*(1,15) = .002, *p* = .969, 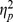
<.01. Finally, as expected, there was an interaction between previous and current congruency, *F*(1,15) = 38.650, *p*<.001, 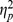 = .72, indicating less interference after incongruent trials (72 ms) than after congruent trials (97 ms). Tests of simple effects revealed that mean RT was longer in cI trials (585 ms) than in iI trials (572 ms), *t*(15) = 4.070, *p* = .001, *η* ^2^ = .52, and longer in iC trials (500 ms) than in cC (488 ms) trials, *t*(15) = 3.487, *p* = .003, *η* ^2^ = .45. No other effects were significant.

As in Experiment 1, we also investigated whether CSEs were influenced by trial *n* – 2 feature repetitions. To do so, we conducted a second repeated-measures ANOVA on mean RT with three within-participants factors: previous congruency (congruent, incongruent), current congruency (congruent, incongruent), and trial *n* – 2 feature repetitions (complete repetitions, complete alternations, target repetitions, distracter repetitions). As in the overall analysis, there was a significant main effect of current congruency, *F*(1,15) = 137.100, *p*<.001, 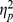 = .90, a significant main effect of previous congruency, *F*(1,15) = 11.821, *p* = .004, 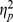 = .44, and a significant interaction between previous congruency and current congruency, *F*(1,15) = 25.331, *p*<.001, 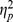 = .63. New to the present analysis, there was a significant main effect of trial *n* – 2 feature repetitions, *F*(3,45) = 13.489, *p*<.001, 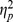 = .47, and a significant interaction between trial *n* – 2 feature repetitions and current congruency, *F*(3,45) = 7.992, *p*<.001, 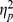 = .35. However, there was neither a two-way interaction between trial *n* – 2 feature repetitions and previous congruency, *F*(3,45) = .674, *p* = .572, 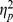 = .04, nor a three-way interaction among trial *n* – 2 feature repetitions, previous congruency, and current congruency, *F*(3,45) = .450, *p* = .718, 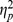 = .03. Thus, trial *n* – 2 feature repetitions did not influence CSEs.

#### Percentage error rate

The percentage error rate data are also presented in Figure 2. Mean percentage error rate was analyzed using a repeated measures analysis of variance (ANOVA) with two factors: previous congruency (congruent vs. incongruent) and current congruency (congruent vs. incongruent). As expected, there was a main effect of current congruency, *F*(1,15) = 6.108, *p* = .026, 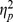 = .29, because mean percentage error rate was higher in incongruent trials (4.6%) than in congruent trials (2.3%). Unlike in Experiment 1, however, no other effects were significant. We note, however, that although the interaction between previous congruency and current congruency was not significant, *F*(1,15) = 1.968, *p* = .181, 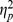 = .12, there was numerically greater interference after congruent trials (2.76%) than after incongruent trials (1.94%), which paralleled the significant results in the RT data. Thus, there was no evidence of a speed-accuracy trade-off.

### Discussion

Using direction words rather than arrows as stimuli, Experiment 2 yielded a significant CSE (though only in response times). This result suggests there is more to the CSE in the prime-probe task than just feature repetition and contingency learning confounds. It also completely rules out an alternative interpretation of the CSE in this task as reflecting feature repetitions made possible by a mental rotation strategy.

### General Discussion

The present study makes three important contributions to the literature on congruency sequence effects (CSEs). First, it shows for the first time that CSEs can be observed in a prime-probe task independent of feature repetition and contingency learning confounds. Second, it shows that this effect can be observed even when participants alternate between two tasks that involve completely different stimulus sets (horizontal versus vertical) and response modes (left hand versus right hand), thereby revealing that the CSE occurs under a less restrictive set of task conditions in the prime-probe task than in the flanker task [23]–[24]. Third, it reveals a highly effective approach for observing CSEs independent of the typical confounds that can easily be adopted by other researchers. These findings have important implications for our understanding of how task context influences behavioral performance in distracter interference tasks.

### CSEs exist independent of the typical confounds in the prime-probe task

As mentioned above, our study provides the strongest evidence to date of CSEs in the prime-probe task independent of feature integration and contingency learning confounds. An important implication of this result is that it breathes new life into the possibility that trial-by-trial adjustments of attention might contribute to CSEs. As reviewed in the Introduction, it has been suggested that control systems sensitive to response conflict [6], expectations regarding upcoming stimulus congruency [5], [27], or negative affect [7] alter the distribution of attention to target and/or distracter stimuli in ways that lead directly to CSEs. Our study was not designed to distinguish among these possibilities. Therefore, future studies should be conducted to investigate whether any of these putative triggers of increased control (conflict, expectancy, or affect) leads to CSEs in the present tasks.

Given previous reports that CSEs in the Stroop, flanker, and Simon tasks often vanish when feature integration and contingency learning confounds are absent [11], [16], it is interesting that our paradigm produced robust CSEs without such confounds. A potential explanation is that the distracter in the prime-probe task is presented before the target, rather than simultaneously with it. This temporal separation may enhance the ability of selective attention to separately modulate target and/or distracter processing. For instance, it may be easier to adjust attention to a distracting prime if it is temporally-separated from a target, relative to when the two are presented simultaneously. Future studies could be conducted to directly test this hypothesis.

A second potential explanation is that 50% of the trials in the prime-probe task were congruent, whereas only 25% of the trials were congruent in the aforementioned Stroop, flanker, and Simon tasks. Employing a low percentage of congruent trials may encourage subjects to enhance target processing and/or suppress distracter processing equally in all trials in order to block out the frequently-conflicting distracter [18], which might reduce the probability of observing CSEs. On the other hand, some work has suggested that CSEs and proportion congruency effects are generally unrelated to each other [19]–[21]. Consistent with this view, Kunde and Wuhr [17] observed a robust CSE in a similar task with only 25% congruent trials. Future studies aimed at distinguishing among these potential explanations could reveal important information about the processes that give rise to CSEs.

It is interesting that our paradigm yielded robust CSEs for another reason: paradigms in which participants alternate between distinct stimulus sets often fail to produce CSEs (with some notable exceptions, [28]). Specifically, many researchers have reported an absence of CSEs when the previous- and current-trial “conflict” types (e.g., Stroop versus Simon) differ [19], [29]–[31], including in the prime-probe task [32]. Our paradigm is different, however, in that both the task – direction discrimination – and the conflict type – direction distracter – never change. Thus, our findings suggest that when participants switch between distinct stimulus sets, CSEs are more likely when the conflict type remains the same than when it changes, in accordance with the suggestions of the researchers cited above.

### CSEs appear under different conditions in the prime-probe and flanker tasks

A second important implication of the present study is that it shows that CSEs can be observed under a less restrictive set of task conditions in the prime-probe task than in the color flanker task [23]. Indeed, unlike in the flanker task [10], [23], we observed CSEs in the prime-probe task even when participants alternated between two tasks involving (a) horizontal versus vertical stimuli and (b) left hand versus right hand responses. Future studies will be needed to determine why CSEs appear under a less restrictive set of task conditions in the prime-probe task than in the flanker task. Whatever the outcome of these studies, an important implication of the present findings is that CSEs appear under a broader range of task conditions in the prime-probe task than in the flanker task. The prime-probe task may therefore be a more versatile and robust tool with which to investigate trial-by-trial adjustments of cognitive control.

### The present methodology can be easily adopted by future researchers

On a related note, a third important implication of the present study is that it reveals a highly effective approach for observing robust CSEs independent of feature integration and contingency learning processes that can easily be adopted by other researchers. Although some researchers have attempted to model the unique contribution of CSEs to behavioral performance when confounds are present [33], such modeling relies heavily on two untested assumptions: (1) that all of the factors contributing to performance are included in the model, and (2) that these factors predict behavioral performance in a linear manner, rather than in a nonlinear interactive fashion (for a discussion of these issues, see Schmidt [15]). By removing confounds instead of modeling them, the present approach more convincingly removes the potential influence of those confounds on CSEs.

Future applications of the present approach might also prove useful for more accurately characterizing how distinct brain regions contribute to CSEs. For example, in line with the conflict monitoring model, evidence from functional magnetic resonance imaging (fMRI) suggests that the anterior cingulate cortex (ACC) signals the presence of response conflict to regions of the dorsolateral prefrontal cortex (DLPFC) that resolve conflict, and that this interaction leads directly to CSEs [6], [12], [34]. Also consistent, temporary lesions to the ACC in humans undergoing neurosurgery appear to eliminate CSEs [35]. However, the paradigms employed in these studies contained feature integration and/or contingency learning confounds. It has therefore been suggested that brain regions posited to underlie conflict processing may instead underlie basic learning and memory processes [15]. Consistent with this possibility, prior work has linked the ACC and DLPFC to basic learning and memory processes [36], including contingency learning processes that are often confounded with CSEs in distracter interference tasks [37]. Future fMRI studies of CSEs using the present methodology could therefore help to clarify the functional contributions of the ACC and DLPFC to producing CSEs.

### Limitations

One potential limitation of the present study is that the method removes only the influence of first-order feature repetitions on CSEs. It is therefore possible that feature repetitions from two or more trials back interacted with conflict-triggered control in trial *n* – 1 to drive CSEs in the present tasks. Consistent with this possibility, Blais and Verguts [38] present a computational model in which feature repetitions from two or more trials back drive CSEs in trial *n*, even when trial *n* repeats no features from trial *n* – 1. The key idea is that conflict-triggered control in trial *n* – 1 leads to a strengthening of associations between the current task set and stimulus and response features from previous trials (which remain active for a few trials after they are presented), thereby increasing CSEs in trial *n* if it contains feature repetitions from two or more trials back. However, Blais and Verguts found no empirical evidence (i.e., from human participants) to suggest that CSEs were influenced by such a “repetition-control” interaction after discarding trials in which features from trial *n* – 1 were repeated. Similarly, we found no evidence to suggest that feature repetitions from trial *n* – 2 influenced CSEs in the present tasks (see Footnotes 1 & 2). Thus, it appears unlikely that “repetition-control” interactions influenced CSEs in the present study.

A second potential limitation of our study is that it does not reveal whether differences in perceptual similarity between the distracter and the target contribute to the CSE. Consistent with this possibility, prior findings from the Stroop and flanker tasks suggest that a CSE might be especially large when the distracter and target stimuli are perceptually similar [39]–[40], which was the case in both of our experiments (i.e., arrows as distracters to arrows in Experiment 1 and location words as distracters to location words in Experiment 2). However, since these previous studies did not control for feature repetition and/or contingency learning confounds, they do not allow a firm conclusion to be drawn. Moreover, some evidence argues against a role for perceptual similarity in driving the CSE, as controlling for feature repetitions and contingency biases in a flanker task has been shown to eliminate the CSE [11], even though the distracters and targets were perceptually identical. Still, perceptual similarity might play some role in driving the CSE. To gain further insight into this issue, future researchers could employ experimental tasks analogous to those in the present study, which do not contain the typical learning and memory confounds. For instance, one could use arrow primes and location word probes, or vice-versa, to determine whether the CSE in the prime-probe task vanishes when the distracter and target are perceptually distinct.

A third potential limitation of the present study is that the method may not adequately control for all learning and memory confounds. For example, in a recent review of the CSE and proportion congruency literatures Schmidt [15] suggested that temporal learning might also contribute to CSEs. According to the *temporal learning hypothesis*, participants develop expectations about *when* they will respond based on recent trial history: they expect to respond quickly following a quick response and slowly following a slow response. In the context of a typical distracter interference task, this hypothesis predicts that participants will expect to respond quickly after a congruent (fast) trial but slowly after an incongruent (slow) trial. As we describe next, this expectation alone could produce a CSE.

How could this expectation lead to a CSE? If the previous trial was congruent, this expectation could lead to a temporary drop in the response threshold relatively early in the current trial (i.e., around the time a response was produced in the prior congruent trial). This would facilitate congruent (fast) responses, because such responses would generally be active enough early in the trial to cross the reduced threshold. However, incongruent (slow) responses would not be facilitated, because a response would not be active enough early in the trial to benefit from the temporarily reduced threshold. The reverse would hold following an incongruent (slow) trial, as the temporary drop in the response threshold would now occur relatively late in the current trial (i.e., around the time a response was produced in the prior incongruent trial). This late drop in the response threshold would facilitate incongruent (slow) responses, because such responses would generally be active enough late in the trial to cross the reduced threshold. In contrast, congruent (fast) responses would not be facilitated, because they would typically be active enough early in the trial to cross the threshold before it drops. For these reasons, responses would be faster when temporal expectations are met (i.e., in cC and iI trials) than when they are violated (i.e., in iC and cI trials), mimicking the pattern predicted by attention adaptation accounts of CSEs (i.e., greater interference after congruent trials than after incongruent trials).

Given these considerations, we acknowledge the possibility that CSEs in the prime-probe task may stem from temporal learning processes, rather than from changes in attention. Consistent with this possibility, temporal learning is well documented in the literature [41]–[42], and previous trial RT influences the magnitude of the congruency effect [43]–[44]. There is little direct evidence, however, that temporal learning drives CSEs, and some prior findings even argue against this view. In particular, first-order CSEs in the Stroop and flanker tasks were absent in two prior studies that employed the present method for removing feature integration and contingency learning confounds, even though a significant congruency effect was observed in each task [10], [22]. Additional studies will therefore be needed to determine the precise circumstances under which temporal learning processes might account, either wholly or in part, for the CSE (see also the related congruency switch account [11]).

### Conclusion

The present study provides one of the most convincing demonstrations to date that CSEs can be observed without feature integration and contingency learning confounds. It also shows that the CSE can be observed under a much less restrictive set of task conditions in the prime-probe task than in the flanker task. As such, the present study provides some of the most encouraging evidence to date that trial-by-trial adjustments of attention might contribute to CSEs independent of the typical confounds. It also reveals an effective approach for observing CSEs independent of these confounds while maximizing the number of trials that can contribute to analyses of CSEs. This approach should facilitate future research on CSEs, thereby allowing future studies to paint a more accurate picture of the psychological and neural mechanisms underlying contextual modulations of behavioral performance in distracter interference tasks.

## Supporting Information

Raw Data S1 **The raw data collected from each participant in Experiments 1 and 2.**
(ZIP)
